# Improvement in Facial Wrinkles Using Materials Enhancing *PPARGC1B* Expression Related to Mitochondrial Function

**DOI:** 10.3390/cimb46060302

**Published:** 2024-05-21

**Authors:** Hyejin Lee, Sanghyun Ye, Juhyun Kim, Seung-Hyun Jun, Nae-Gyu Kang

**Affiliations:** LG Household & Health Care (LG H&H) R&D Center, Seoul 07795, Republic of Korea; hellohj1223@lghnh.com (H.L.); shye123@lghnh.com (S.Y.); juhyunkim@lghnh.com (J.K.)

**Keywords:** skin aging, mitochondria function, wrinkle improvement, cosmetics

## Abstract

Skin aging is an unavoidable natural phenomenon caused by intrinsic and extrinsic factors. In modern society, the pursuit of a wrinkle-free and aesthetically appealing face has gained considerable prominence. Numerous studies have aimed at mitigating the appearance of facial wrinkles. Antiaging research focused on regulating the function of mitochondria, the main reactive oxygen species-generating organelles, has been extensively conducted. In this study, we investigated the correlation between facial wrinkles and the expression of *PPARGC1B*, considering the association of this gene with mitochondrial function, to identify its potential as a target for exploring antiaging cosmetic materials. We elucidated the role of *PPARGC1B* in the skin and identified five bioactive materials that modulated its expression. The effectiveness of these materials was verified through in vitro experiments on human dermal fibroblasts. We prepared cosmetic formulations incorporating the five materials and confirmed their ability to enhance dermal collagen in three-dimensional skin models and reduce facial wrinkles under the eyes and nasolabial fold areas in human subjects. The study findings have significant implications for developing novel antiaging cosmetic formulations by reinforcing mitochondrial functions.

## 1. Introduction

As the outermost layer of the body, the skin is constantly exposed to the external environment. Skin aging can be categorized into intrinsic aging, which occurs due to endogenous factors within the cells, and extrinsic aging, influenced by external factors. As aging progresses, a series of events occur that result in a decline in the function of cells, the basic structural units, and impaired metabolic interactions between cells, leading to a gradual decline in the overall condition of the skin [1]. Aged skin is characterized by wrinkles, loss of elasticity, and a rough texture. Skin aging is a natural phenomenon that progresses over time. In modern society, with increased interest in skin care, the desire to maintain young and attractive skin is common even at early ages. Middle-aged and older people, in particular, exhibit a keen interest in diminishing wrinkles, as it profoundly impacts their perceived attractiveness and cognitive age [2]. Despite significant advances in antiaging dermatological procedures and home care devices [3,4,5], a demand exists for safe and effective antiaging cosmetics in daily skincare routines.

According to the “Free radical theory of aging” proposed by Harman [6], elevated levels of reactive oxygen species (ROS) are pivotal in progressively damaging cellular structures, including proteins, lipids, DNA, and RNA, thereby inducing impairment of cellular function and ultimately contributing to the aging process [7,8]. ROS are inevitable byproducts of cellular respiration. Although ROS serve as beneficial signaling molecules in cellular metabolism at appropriate levels, excessive ROS production can be deleterious [9,10]. Mitochondria, the primary organelles responsible for ATP production through oxidative phosphorylation (OXPHOS), are the major source of ROS [7]. Notably, with its high cellular turnover and substantial energy requirements, the skin represents an organ in which efficient ATP generation via mitochondrial respiration is critical for maintaining physiological functions. However, this process inevitably leads to oxidative stress due to surplus ROS production, which can directly impair mitochondrial function [11]. As mitochondria are pivotal organelles involved in energy homeostasis and ROS generation, their tight lifespan regulation and constant renewal through processes such as mitochondrial biogenesis and mitophagy are paramount [12]. Several studies have highlighted the significance of age-related alterations in the number of mitochondria and mitochondrial DNA (mtDNA) in various organs, including skeletal muscle [13,14] and heart [15]. Age-related decline includes alterations in the mitochondrial copy number and their functional attributes, such as volume, density, and oxidative capacity, which are intimately associated with mitochondrial biogenesis [12,16]. 

Mitochondrial biogenesis in mammalian cells is regulated by the transcriptional family of peroxisome proliferator-activated receptor γ (PPARγ) coactivator-1 (PGC-1) [17]. Among the three subtypes of the PGC-1 family (PGC-1α, PGC-1β, and PRC), PGC-1α is widely recognized as the major regulator of mitochondrial biogenesis. PGC-1α undergoes phosphorylation and acetylation, which are mediated by two distinct cellular energy sensors, namely AMP-activated protein kinase (AMPK) and sirtuin 1 (SIRT1), respectively. After these post-translational modifications, PGC-1α affects mitochondrial biogenesis via interaction with nuclear respiratory factor 1 (NRF-1) and estrogen-related receptor alpha (ERRα), an orphan nuclear hormone receptor [18]. Although PGC-1α has garnered attention as a pivotal regulator of mitochondrial biogenesis, PGC-1β has been recognized as an important regulator and performs a similar role in the mitochondria. Small interfering RNA (siRNA)-mediated silencing of endogenous PGC-1β results in the inhibition of mitochondrial function-associated genes, such as *COXI*, *COX7b*, and *Tfb2m* [19]. Adenovirus-mediated overexpression of PGC-1β leads to the induction of genes involved in mitochondrial biogenesis, such as *NRF-1*, *ERRα*, and *Tfam* [19,20,21]. Numerous studies have been conducted to elucidate the functions of the PGC-1 family members and their relationship with mitochondria. Although extensive efforts have been devoted to explore their roles in different cell types, including muscle cells, which perform specialized functions for substantial energy consumption, direct functional studies on human skin cells and their usage as targets for the discovery of novel antiaging cosmetic ingredients are scarce. In an investigation on the potential of the PGC-1 family as a target for antiaging cosmetics, the efficacy of alleviating wrinkles by restoring mitochondrial markers, namely PGC-1α, Tom 20, and COX IV, was demonstrated in UVB-induced photoaged skin [22]. Although this study was conducted using mouse models, the results support the hypothesis that anti-wrinkle materials can be discovered using human skin by analyzing their effects on the regulation of the PGC-1 family.

A genome-wide association analysis to identify genetic loci associated with facial wrinkles in the Korean population unraveled variants of *PPARGC1B* that encode PGC-1β as a significantly linked locus [23]. In this study, we examined a library of bioactive materials, suitable as cosmetic ingredients, to identify substances possessing the ability to enhance the expression of *PPARGC1B*, the gene encoding PGC-1β. The term ‘material’ comprehensively refers to various raw materials or ingredients such as oils, surfactants, antioxidants, and bioactive substances used in the cosmetics industry [24]. In previous research related to the cosmetics industry, cosmetics formulations that improve the condition of the skin have been developed through research on discovering and applying bioactive materials that have a beneficial effect on various cells of the skin and applying them to the skin [25,26,27]. We evaluated the efficacy of various bioactive materials including niacinamide (NAM), camphor (CAM), sodium mannose phosphate (SMP), tranexamic acid (TNA), and pyridoxine hydrochloride (P-HCl) both in vitro and ex vivo. Subsequently, a complex formula containing the five aforementioned active materials was tested on human subjects in a clinical trial. Our study provides evidence for the positive relationship between the enhancement of mitochondrial function through the regulation of the mitochondria-related gene *PPARGC1B* and the inhibition of human skin aging, particularly for facial wrinkles.

## 2. Materials and Methods

### 2.1. Cell Culture and Preparation

The human dermal fibroblast cell line Hs68 (ATCC, Manassas, VA, USA) was cultured in Dulbecco’s modified Eagle medium (DMEM; Gibco, Waltham, MA, USA) supplemented with 10% (*v*/*v*) fetal bovine serum (FBS; Gibco, Waltham, MA, USA) and penicillin-streptomycin (Gibco, Waltham, MA, USA) at 37 °C in an atmosphere with 5% CO_2_. 

NAM (pyridine-3-carboxamide), CAM (1,7,7-trimethylbicyclo [2.2.1]heptan-2-one), P-HCl, TNA, and retinol were purchased from Sigma-Aldrich (St. Louis, MO, USA). Agefinity™ containing SMP was purchased from Givaudan (Vernier, Canton of Geneva, Switzerland).

### 2.2. Hs68 Cell Transfection with siRNA for RNAi Experiments

Three types of *PPARGC1B* siRNAs and one negative control siRNA (siNC) were purchased from Bioneer (Daejeon, Korea): AccuTarget™ Genome-wide Predesigned siRNA (SDH-1001): siRNA No133522-1, 133522-2, and 133522-3 for *PPARGC1B* and AccuTarget™ Negative Control siRNA (SN-1003). These siRNAs were dissolved in diethyl pyrocarbonate-treated distilled water to yield a 100 µM concentration. Hs68 cells were plated at 3 × 10^5^ cells/cm^2^ in a six-well plate and transfected with 100 nM siRNAs using Lipofectamine^®^ 2000 (Thermo Fisher Scientific, Waltham, MA, USA) following the manufacturer’s instructions. After 6 h of transfection, the cells were washed with 1X phosphate-buffered saline (PBS), and fresh DMEM containing 10% FBS was added to the wells. After incubation for 16 h, the cells were collected for subsequent analyses. *PPARGC1B* knockdown efficiencies were measured using real-time quantitative PCR (RT-qPCR) compared to siNC.

### 2.3. RNA Extraction and RT-qPCR

RNA was extracted from Hs68 cells seeded in six-well plates (3 × 10^5^ cells/well) using an RNeasy mini kit (Qiagen, Hilden, Germany). The RNA concentration and purity were measured using a NanoDrop 2000 spectrophotometer (Thermo Fisher Scientific, Waltham, MA, USA). One microgram of RNA was reverse-transcribed into cDNA using a cDNA synthesis kit (Philekorea, Seoul, Korea) following the manufacturer’s protocol, and RT-qPCR was performed using the StepOnePlus^®^ Real-Time PCR System (Applied Biosystems, Waltham, MA, USA). The following TaqMan probes were used for RT-qPCR: PPARGC1B (Hs00993805_m1), NRF1 (Hs00602161_m1), TFAM (Hs00273372_s1), COL1A1 (Hs00164004_m1), COL4A1 (Hs00266237_m1), COL4A2 (Hs05006309_m1), ELN (Hs00355783_m1), HAS2 (Hs00193435_m1), HAS3 (Hs00193436_m1), MMP1 (Hs00899658_m1), MMP9 (Hs00957562_m1), and human GAPDH (Hs02786624_g1) endogenous control (Thermo Fisher Scientific, Waltham, MA, USA).

### 2.4. UV Irradiation

After seeding and overnight culture, the medium in the wells with adhered Hs68 cells was replaced with 1X PBS. The cells were irradiated once with UVB using a BIO-SUN irradiation system (Vilber Lourmat, Marne-la-Valle’e, France), with an irradiation intensity, time, and distance of 30 mJ/cm^2^, 10–15 s, and 25 mm, respectively. Thereafter, Hs68 cells were treated with fresh serum-free medium containing active materials.

### 2.5. Flow Cytometry Analysis of Mitochondrial Membrane Potential (ΔΨm)

Hs68 cells were seeded into six-well plates at a density of 2 × 10^5^ cells/well and cultured overnight at 37 °C in an atmosphere with 5% CO_2_. Before treatment with the active materials, cells in the UVB treatment group were exposed to UVB, as mentioned in Section 2.4. Twenty-four hours after the treatment, the cells were trypsinized and washed three times with 1X PBS. For staining, 500 nM of MitoTracker™ Red CMXRos (Thermo Fisher Scientific, Waltham, MA, USA) was added to the wells, and cells were incubated at 37 °C for 30 min in the dark. The unbounded dye was removed with three washes with 1X PBS. Fluorescence signals were analyzed using a CytoFLEX Flow Cytometer (Beckman Coulter, Brea, CA, USA).

### 2.6. ATP Assay

The ATP levels in Hs68 cells treated with active materials were measured using CellTiter-Glo^®^ Luminescent Cell Viability Assay (Promega, Madison, WI, USA) following the manufacturer’s instructions. Briefly, 1.5 × 10^4^ cells/well were seeded in black 96-well plates and cultured overnight at 37 °C in an atmosphere with 5% CO_2_. Before treatment with the active materials, cells in the UVB treatment group were exposed to UVB, as mentioned in Section 2.4. Twenty-four hours after the treatment, the plates were equilibrated for 30 min at room temperature. Next, 100 μL of the CellTiter-Glo^®^ reagent was added to each well and mixed for 2 min to induce cell lysis. After incubation at room temperature for 10 min, the luminescence signal was recorded on a Varioskan™ LUX multimode microplate reader (Thermo Fisher Scientific, Waltham, MA, USA).

### 2.7. Procollagen Type I C-Peptide Assay

Procollagen type I C-peptide (PIP) secreted from Hs68 cells was quantified with a Procollagen Type I C-peptide (PIP) EIA Kit (MK101; Takara; Shiga, Japan) following the manufacturer’s instructions. Briefly, 2 × 10^4^ cells/well were seeded in a 24-well plate and cultured overnight at 37 °C in an atmosphere with 5% CO_2_. Twenty-four hours after treatment with active materials, the culture supernatant was collected to measure the secreted human procollagen I alpha 1. The amount of PIP in each sample was normalized against the total protein content, which was measured using the Pierce™ BCA Protein Assay Kit (Thermo Fisher Scientific, Waltham, MA, USA). 

### 2.8. Reconstructed Three-Dimensional (3D) Human Skin

The reconstructed 3D human skin model Neoderm^®^-ED was purchased from Tego Science (Seoul, Korea) and maintained and cultured following the manufacturer’s instructions. For the experiment, two types of test cream formulations were applied to the 3D skin. In the first formulation (cream 1), 3% NAM, 0.001% CAM, 0.06% Agefinity™, 0.5% TNA, and 0.06% P-HCl were added to O/W type vehicle cream formulation. The vehicle formulation comprised cetyl stearyl alcohol, stearyl alcohol, glyceryl stearate, PEG-40 stearate, beeswax, C14-22 alcohols, C12-20 alkyl glucoside, lecithin, caprylic/capric triglyceride, squalene, cyclopentasiloxane, cyclohexasiloxane, dimethicone/vinyl dimethicone crosspolymer, isocetyl myristate, dipropylene glycol, glycerin, betaine, 1,2-hexanediol, EDTA-3Na, xanthan gum, carbomer (2-propenoic acid, polymer with 2,2-bis(hydroxymethyl)propane-1,3-diol 2-propenyl ether), tromethamine, and distilled water. In the second formulation (cream 2), 0.1% retinol was added to the first formulation. The same vehicle formulation without bioactive materials was applied as the control. Briefly, 30 μL each of vehicle, cream 1 and cream 2 were applied on top of the reconstructed 3D human skin model, and incubated for 48 h at 37 °C in an atmosphere with 5% CO_2_. Subsequently, the cultured 3D skin model was fixed with 4% paraformaldehyde, embedded in a paraffin block, and stained with Masson’s trichrome stain. The stained images of sectioned 3D human skin model were acquired using the EVOS™ FL Auto2 Imaging System (Thermo Fisher Scientific, Waltham, MA, USA). Dermal collagen area and epidermal thickness were analyzed using the Image J Software (NIH, Bethesda, MD, USA).

### 2.9. Human Clinical Test

This study was approved by the ethics committee of LG H&H Institutional Review Board (LGHH-20211014-AA-05-01, 15 October 2021). Eleven healthy Korean women, aged 40 to 62 years (mean age, 50.1 years), were recruited. The possible side effects were explained to the women, and informed consent for participation in the clinical trial was obtained. Pregnant women or people undergoing dermatological procedures were excluded from the test. The test was performed in a half-face and double-blind manner. The test formulation containing 3% NAM, 0.5% TNA, 0.1% pyridoxine P-HCl, 0.05% CAM, 0.06% Agefinity™, and 0.1% retinol was topically applied on the right side of the face twice daily for 4 weeks. For the control, a formulation containing only 0.1% retinol was topically applied on the other side of the face. All types of creams used in this study were produced at the LG H&H R&D center following our protocol. 

Before measurements, all participants were asked to wash their face thoroughly and then rest for at least 20 min in a room with controlled relative humidity (45 ± 5%) and temperature (22 ± 2 °C). Wrinkles on the face were measured using an Antera 3D camera (Miravex, Dublin, Ireland). Changes in fine wrinkles under the eyes were analyzed using the texture Ra parameter, and nasolabial folds were analyzed using the fold length parameter in the Antera 3D program.

### 2.10. Statistical Analysis

Data are presented as mean values ± SD derived from at least three independent experiments. Statistical analysis of data was performed using Student’s *t*-test and analysis of variance (ANOVA). A *p*-value less than 0.05 (** *p* < 0.05, * *p* < 0.1) indicated a significant difference.

## 3. Results and Discussion

### 3.1. Functional Study of Wrinkle-Related Gene, PPARGC1B, in Hs68 Cells

PGC-1β actively participates in mitochondrial biogenesis and respiration through its direct interaction with NRF-1 and estrogen-related receptor α (ERRα) [19]. The involvement of several nuclear transcription factors, including NRF-1, NRF-2, ERRα, and mitochondrial transcription factor A (TFAM), in mitochondrial biogenesis and function has been reported [28,29,30]. Despite numerous hypotheses on the connection between mitochondrial function and skin aging [31], studies exploring the function of PGC-1β in skin cells are scarce. To address this gap in knowledge, we conducted a preliminary investigation into the correlation of PGC-1β with the expression of skin extracellular matrix (ECM) genes and mitochondrial function by siRNA-mediated knockdown of *PPARGC1B*. 

**Figure 1:**
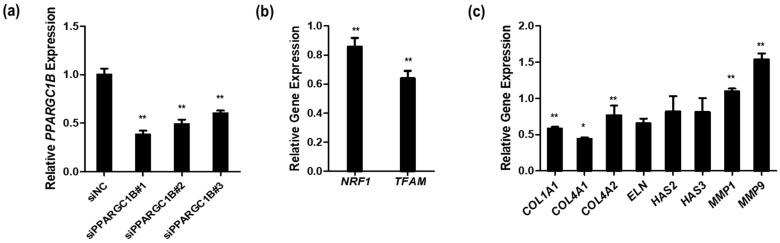
Effects of *PPARGC1B* knockdown in human dermal fibroblasts. (**a**) Downregulation of *PPARGC1B* expression using three siRNAs compared to negative control siRNA (siNC); (**b**) Expression of *NRF1* and *TFAM*, associated with mitochondrial biogenesis, in *PPARGC1B*-knockdown cells; (**c**) Expression of genes associated with human skin extracellular matrix components in *PPARGC1B*-knockdown cells. Bars indicate standard deviation. ** *p* < 0.05, * *p* < 0.1; Student’s *t*-test.

To effectively inhibit the expression of *PPARCG1B*, we confirmed the reduced *PPARGC1B* expression in si*PPARGC1B*-transfected cells compared to that in negative control siNC-transfected cells. We analyzed the effect of *PPARGC1B* knockdown on genes related to both mitochondria and ECM, such as *NRF1*, *TFAM*, *COL1A1*, *ELN*, *HAS2*, and *MMP1*. We confirmed the inhibition rate of *PPARGC1B* and selected the siRNA, from among the three types available, with the highest transfection and knockdown efficiency. The selected siRNA (#1) reduced the expression of *PPARGC1B* by up to 62% (Figure 1a) compared to negative control siNC, without any discernible effect on cell viability. Subsequent analysis of gene expression related to mitochondrial function in cells with reduced *PPARGC1B* expression treated with siPPARGC1B revealed a significant reduction of 14% and 36% in the expression of *NRF1* and *TFAM*, respectively (Figure 1b). Among the ECM genes, the expression of *COL1A1*, *COL4A1*, *COL4A2*, *ELN*, *HAS2*, and *HAS3* was downregulated in the siPPARGC1B-treated cells. 

Conversely, the expression of *MMP1* and *MMP9*, factors involved in collagen decomposition, was increased in siPPARGC1B-treated cells (Figure 1c). Previous studies have examined the effect of PGC-1 on skin aging from a photoaging perspective. ROS production near the mitochondrial inner membrane, where mtDNA is located, under UV exposure, increases the likelihood of mtDNA mutation and impairment of mitochondrial function. These changes contribute to the typical signs of aging resulting from declining cellular function [22,32]. The dermis of healthy, beautiful-looking skin contains a collagen-rich ECM. As aging progresses, the occurrence of wrinkles can be attributed to either the decomposition of ECM components or a reduction in their production capacity [33]. In addition to these previous findings, we unraveled a direct connection between decreased expression of *PPARGC1B*, essential for sustaining mitochondrial function, and alterations in the expression of ECM genes that affect skin aging.

### 3.2. Screening Active Materials That Regulate PPARGC1B Expression

We observed a significant downregulation in the expression of ECM factors in human dermal fibroblasts after *PPARGC1B* knockdown, whereas the expression of factors involved in ECM decomposition was increased. Based on these findings, we propose that upregulating the expression of *PPARGC1B* may be a viable strategy to mitigate ECM degradation associated with skin aging.

In terms of mitochondrial metabolism, PGC-1β is crucial in glycolysis and OXPHOS. Deficiency of PGC-1β leads to impaired oxidative metabolism, which is compensated by an increase in glycolytic activity [34]. Supplementation of niacin, a key nutrient in ATP production in skeletal muscles, upregulates the expression of *PPARGC1A* and *PPARGC1B*, ultimately promoting fatty acid utilization within the muscles [35]. In this study, we aimed to identify cosmetic materials with the ability to regulate *PPARGC1B* expression.

**Figure 2:**
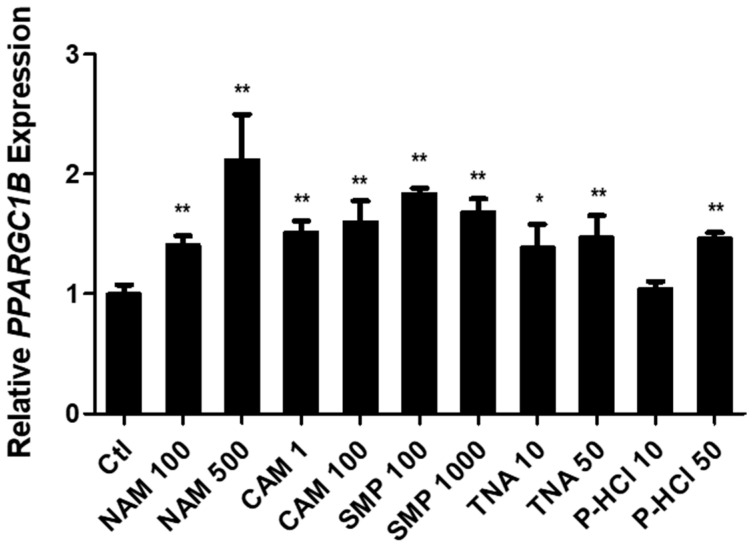
Screening materials with the ability to upregulate *PPARGC1B* expression. Niacinamide (NAM), camphor (CAM), Agefinity™ (SMP), tranexamic acid (TNA), and pyridoxine HCl (P-HCl) increased the expression of *PPARGC1B* at the mentioned concentrations. Bars indicate standard deviation. ** *p* < 0.05, * *p* < 0.1; Student’s *t*-test.

Among the various materials tested, we observed a significant upregulation of *PPARGC1B* expression in response to NAM, a derivative of vitamin B3 that is commonly used as a skin-whitening ingredient in the cosmetics industry. Specifically, treatment with 100 and 500 μg/mL NAM resulted in a 1.41- and 2.12-fold increase in *PPARGC1B* expression, respectively. CAM showed a 1.52- and 1.60-fold increase at 1 and 100 μg/mL, respectively. Agefinity™, containing SMP, showed a 1.84- and 1.68-fold increase in *PPARGC1B* expression at 100 and 1000 μg/mL, respectively. Similarly, TNA showed a 1.39-, 1.47-fold increase in *PPARGC1B* expression at 10 and 50 μg/mL, respectively. Finally, P-HCl exhibited a 1.46-fold increase in *PPARGC1B* expression at 50 μg/mL (Figure 2).

In addition to the aforementioned five materials, we confirmed the effects of other reported wrinkle improvement-related materials on *PPARGC1B* expression. *PPARGC1B* expression was enhanced by retinol (10 μM, *p* < 0.05), hydroxypinacolone retinoate (HPR; 20 μM, *p* < 0.05), bakuchiol (1 μM, *p* < 0.05), adenosine (10 μg/mL, *p* < 0.05), L-hydroxyproline (10 μg/mL, *p* < 0.1), Matrixyl-3000 (Sederma, Le Perray en Yvelines, France; 100 μg/mL, *p* < 0.05), and Panax ginseng root protoplasts (PGRP; 1000 μg/mL, *p* < 0.05) and was reduced by phloretin (10 μg/mL, *p* < 0.05) and oryzanol (1 μg/mL, *p* < 0.05) (Figure S1).

### 3.3. Antiaging Effect of PPARGC1B Expression-Regulating Materials In Vitro

#### 3.3.1. Recovery of UVB-Induced Decrease in ΔΨm

Mitochondria are pivotal in the skin owing to its energy requirements, such as during cell signaling, wound healing, pigmentation, vasculature homeostasis, and hair growth, and mitochondrial dysfunction is closely related to skin aging [31]. Mitochondria, a key organelle involved in ROS generation and apoptosis [36], can be evaluated by assessing ΔΨm as a surrogate marker. ΔΨm, generated via the proton pump activity, is crucial for energy storage during OXPHOS and maintaining mitochondrial homeostasis by selectively eliminating dysfunctional mitochondria. Additionally, it is vital in ensuring healthy mitochondrial function, cellular health, and viability [37]. 

**Figure 3:**
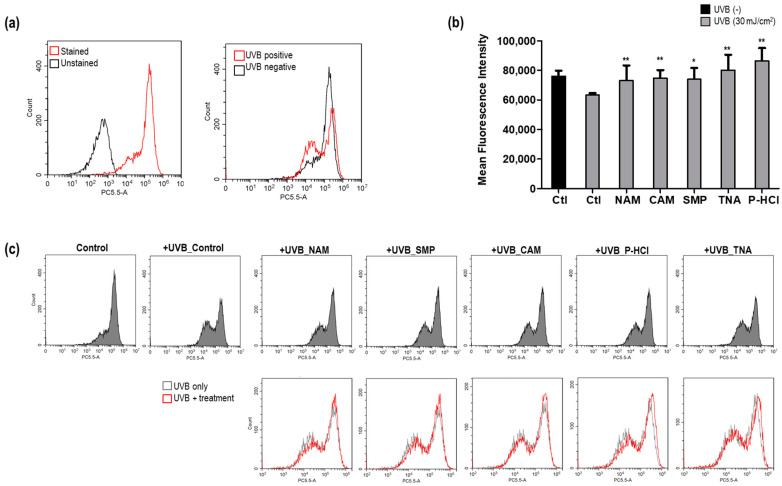
Analysis of mitochondrial membrane potential (ΔΨm) recovery effects of *PPARGC1B* expression-regulating materials under UVB-irradiated conditions. (**a**) Effect of UVB irradiation on ΔΨm in human dermal fibroblasts; (**b**) recovery of ΔΨm decreased by UVB using five *PPARGC1B* expression-upregulating materials; (**c**) comparison of FACS signal plots before and after treatment with each material under UVB irradiation. Bars indicate standard deviation. ** *p* < 0.05, * *p* < 0.1; Student’s *t*-test.

To evaluate ΔΨm, we used MitoTracker™ Red CMXRos, a cationic dye that accumulates in the mitochondrial membrane of live cells depending on the membrane potential [38]. First, using flow cytometry, we determined the ΔΨm level of Hs68 cells irradiated with 30 mJ/cm^2^ UVB, employing the MitoTracker™ Red CMXRos dye [39]. Remarkably, UVB irradiation significantly reduced ΔΨm by approximately 20% compared to nonirradiated cells (Figure 3a). Alterations in ΔΨm levels following a specific stimulus often indicate gain or loss changes in mitochondrial function [39]. We validated that UVB exposure resulted in the loss of mitochondrial function, as manifested by the reduction in ΔΨm.

Next, we investigated the effect of the five materials that regulated *PPARGC1B* expression to confirm the recovery of a UVB-induced decrease in ΔΨm. Upon treatment with 100 μg/mL NAM, 10 μg/mL CAM, 1000 μg/mL SMN, 50 μg/mL TNA, and 10 μg/mL of P-HCl, the ΔΨm level increased by 12.3% (80% to 92.3%), 14.5% (80% to 94.5%), 13.7% (80% to 93.7%0, 21.3% (80% to 101.3%), and 29.3% (80% to 109.3%) compared with that in control irradiated with UVB, respectively (Figure 3b,c). These materials function as enhancers of mitochondrial function under stress conditions, such as UV irradiation.

#### 3.3.2. Recovery of UVB-Induced Decrease in ATP Synthesis and Promotion of ATP Synthesis

PGC-1β is a key regulator of mitochondrial metabolism, and PGC-1β deficiency reduces the ATP production capacity in immune cells [34]. In the skin, ATP is a form of cellular energy supplied by the mitochondria. ATP production is reduced during mitochondrial damage during the intrinsic and extrinsic skin aging processes [40]. Therefore, following the evaluation of ΔΨm, we investigated the effect of the five active ingredients that increased the expression of *PPARGC1B* on ATP production. 

**Figure 4:**
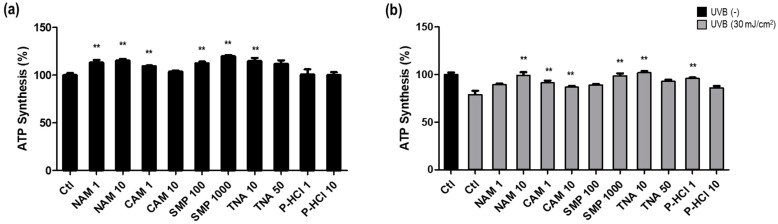
Analysis of ATP synthesis and recovery effects of *PPARGC1B* expression-regulating materials under UVB irradiation. (**a**) Increased ATP synthesis upon treatment with *PPARGC1B* expression-regulating materials; (**b**) recovery of ATP synthesis upon treatment with *PPARGC1B* expression-regulating materials under UVB irradiation. Bars indicate the standard deviation. ** *p* < 0.05; Student’s *t*-test.

We treated the cells with the five active ingredients at two concentrations each under the following conditions: non-UVB irradiation and 30 mJ/cm^2^ UVB irradiation. First, we confirmed that ATP production increased significantly by the most active ingredient even in the absence of damage-inducing stress such as UVB irradiation. Upon treatment with 10 μg/mL NAM, 1 μg/mL CAM, 1000 μg/mL SMP, 10 μg/mL TNA, and 1 μg/mL P-HCl, the ATP levels were increased by 15.4%, 9.6%, 19.8%, 14.8%, and 4.2%, respectively, compared with that in the non-treated control (Figure 4a). When cells were damaged by UVB irradiation, the ATP production ability was decreased by 20.9%. The reduced ATP production ability was recovered when the cells were cultured with the five active ingredients after UVB irradiation. Upon treatment with 10 μg/mL NAM, 1 μg/mL CAM, 1000 μg/mL SMP, 10 μg/mL TNA, and 1 μg/mL P-HCl, ATP levels showed recovery by 20.3%, 12.2%, 19.5%, 22.8%, and 17%, respectively, compared with that in the untreated control irradiated with UVB alone (Figure 4b). These results imply that the five active ingredients that regulate *PPARGC1B* expression promote ATP synthesis and can restore ATP synthesis when mitochondrial damage occurs during skin aging.

#### 3.3.3. Effect of Enhanced Type I Procollagen Synthesis 

The five tested materials affected the ΔΨm, which indicates mitochondrial function. Collagen and mtDNA damage contributes to increased ROS production, particularly during aging, including photoaging [41]. Collagen, the major structural protein in the skin dermis, is essential for maintaining skin health. Its production declines with intrinsic aging, associated with chronological age, or with extrinsic aging, caused by external stimuli, leading to a reduction in its quantity and quality [42]. This decline results in a reduction in dermal thickness over time and skin wrinkling due to a loss in elasticity and flexibility [43]. Therefore, numerous efforts are underway to promote collagen synthesis for improving and preventing facial wrinkles. Collagen is consumed or topically applied along with materials that promote collagen synthesis.

**Figure 5:**
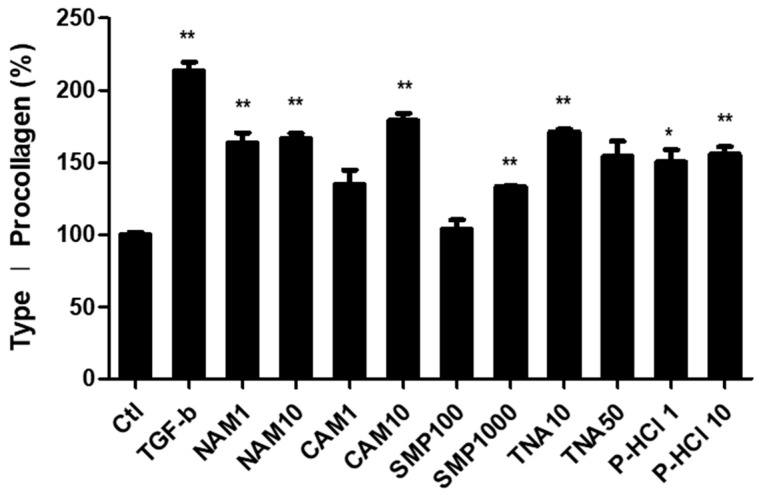
Analysis of changes in type I procollagen synthesis in human dermal fibroblasts. Significant increase in collagen synthesis in groups treated with the *PPARGC1B* expression-regulating materials at least at one of the selected concentrations. Bars indicate standard deviation. ** *p* < 0.05, * *p* < 0.1; Student’s *t*-test.

To date, approximately 28 members of the collagen superfamily have been identified [44]. Among them, three types of collagen are primarily present in the adult skin. Type I collagen accounts for 85–90%, type III collagen accounts for 8–11%, and type V collagen accounts for 2–4% of the total collagen [45]. Considering that type I collagen is the main subtype in the human skin, we investigated the effect of the five active materials on promoting type I collagen synthesis by enhancing mitochondrial function through the upregulation of *PPARGC1B* expression and ΔΨm. The cells were treated with each of the five active ingredients at two concentrations, and all five ingredients significantly promoted collagen synthesis, at least at one of the concentrations used. Treatment with 10 μg/mL NAM, 10 μg/mL CAM, 1000 μg/mL SMN, 50 μg/mL TNA, and 10 μg/mL P-HCl resulted in 62.8%, 74.2%, 33.2%, 64.8%, and 60.8% increases in secreted type I procollagen levels compared with those in the control, respectively (Figure 5). These findings indicate that the five active materials selected in this study are promising candidates for improving wrinkles by enhancing mitochondrial function and promoting collagen synthesis.

### 3.4. Dermal Collagen Increases Efficacy of the Selected Materials in 3D Skin Equivalents 

Although two-dimensional (2D) monolayer cell culture systems offer some advantages in understanding molecular mechanisms, such as molecular signaling, cellular morphology, and the effects of active materials on protein synthesis and gene expression, they fail to fully replicate the 3D in vivo environment [46,47]. Therefore, the development of 3D cell culture systems is progressing steadily, with bioengineered human skin equivalents incorporating human skin cells and ECM components being utilized in skin research [48]. 

**Figure 6:**
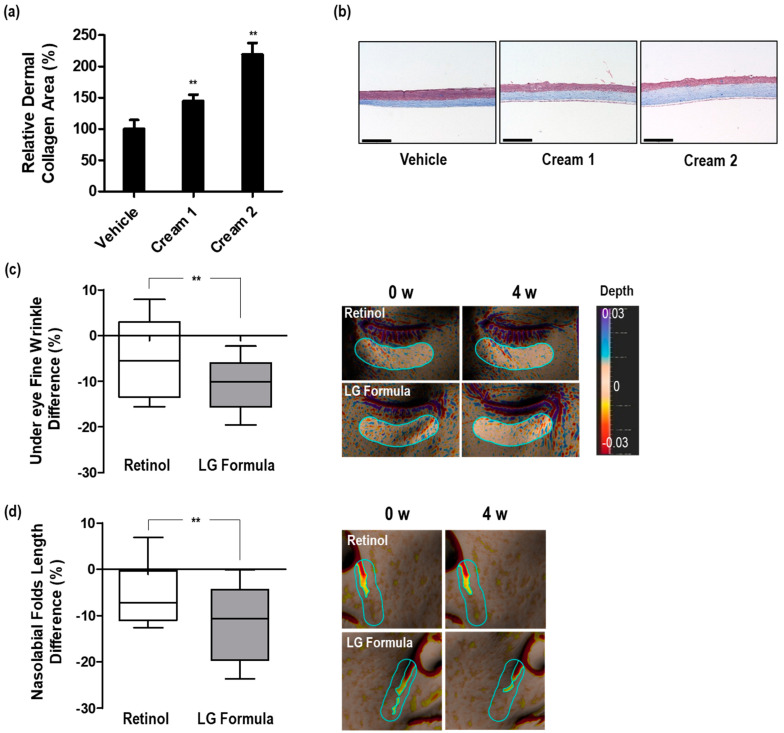
Enhancement of dermal collagen in 3D skin equivalent and facial wrinkle improvement in human subjects by treatment with formulations containing *PPARGC1B* expression regulating materials. (**a**) Effect of two cream formulations on increasing dermal collagen area in 3D skin equivalent; (**b**) representative images of cross sections of 3D skin equivalent; scale bar = 275 μm; (**c**) comparison of the undereye fine wrinkle improvement rate between 0.1% retinol treated and LG formula treated groups after 4 weeks; (**d**) comparison of the nasolabial fold length improvement rate between 0.1% retinol treated and LG formula treated groups after 4 weeks. Each representative image was captured using an Antera 3D camera before and 4 weeks after treatment. Bars indicate standard deviation. ** *p* < 0.05; Student’s *t*-test.

To examine the efficacy of complexes composed of the five active materials, we prepared two formulations of creams and utilized a reconstructed 3D human skin model (Neoderm^®^-ED). Cream 1 comprised only the five active materials (3% NAM, 0.001% CAM, 0.06% SMP, 0.5% TNA, and 0.06% P-HCl) in a vehicle formulation. Cream 2 represented a formulation closer to the final cosmetic product in which 0.1% retinol was added to cream 1. Vehicle, cream 1, and cream 2 were applied to the reconstructed 3D human skin model for 48 h, and the relative ratio of dermal collagen area exhibited a significant increase of 44.6% and 119.1% in the two cream formulations, respectively, compared with the vehicle formulation (Figure 6a). This enhancement in the dermal collagen area, visualized as a blue stain in histological analysis, was also confirmed visually (Figure 6b). No statistically significant changes in the total epidermal thickness were observed (Figure S2). The epidermis is a tissue where turnover occurs actively; hence, limited treatment with substances for 48 h may be considered insufficient to show a dramatic increase in 3D skin models. These findings substantiate the potential of the five active materials in stimulating dermal collagen synthesis when incorporated into cosmetic formulations, not only in a 2D cell culture but also in a 3D human skin model that more closely mimics human skin physiology. Adding retinol, a recognized wrinkle-improving agent, further amplified the efficacy of collagen synthesis promotion, suggesting its potential for wrinkle improvement.

### 3.5. Improvement in Skin Wrinkles by LG Formula-Containing Materials That Increased PPARGC1B Expression

Next, we conducted an in vivo investigation to assess the efficacy of a complex formulation containing the five active materials in improving facial wrinkles. Based on the results of the 3D human skin model test, we designated cream 2 as “LG Formula” and evaluated its effects on wrinkle appearance in vivo. Two creams were tested: a cream containing only 0.1% retinol and another supplemented with 0.1% retinol and the five active materials (LG Formula). Each half of the subjects’ faces was treated with one of the creams twice daily. As expected, after 4 weeks of treatment, the appearance of two types of wrinkles, namely fine wrinkles under the eyes and nasolabial folds, indicated significant improvement (Figure 6c,d). Fine wrinkles, observed in the early stages of facial aging, primarily around the eyes, were evaluated using the roughness (Ra) parameter obtained from skin texture measurements using an Antera 3D camera. Ra is widely used as an indicator to quantify microscopic irregularities on the skin surface, which correspond to fine wrinkles [49,50]. Nasolabial folds, deep skin folds extending from the sides of the nose to the corners of the mouth, were selected as representative aging-associated deep wrinkles. Unlike fine wrinkles that emerge early, nasolabial folds tend to lengthen and deepen over time due to cumulative facial expression and dynamic movements. The presence of nasolabial folds is closely associated with perceived age, often making individuals appear older than their actual age [51]. Consequently, deep nasolabial folds are commonly addressed through dermatological procedures such as hyaluronic acid fillers [52]. In this study, the folded length, commonly employed as a parameter for evaluating nasolabial folds using the Antera 3D camera, was utilized for evaluating the efficacy of the treatments [53].

The topical application of the LG Formula containing retinol and the five active materials for 4 weeks resulted in a noticeable improvement of 10.4% and 11.0% in fine wrinkles under the eyes and deep wrinkles of nasolabial folds, respectively (Figure 6c,d). Notably, this improvement rate was significantly higher than the improvement rates of 4.3% and 5.7% observed when applying 0.1% retinol alone to the same areas (Figure 6c,d). While it is difficult to make direct comparisons with previous studies owing to differences in research conditions, such as methods used for analysis and study subjects, previous research has also demonstrated significant improvements in wrinkle appearance after 4 weeks of retinol application to the face [54,55]. For example, the topical application of a 0.1% retinol preparation for 4 weeks resulted in a 27.93% reduction in wrinkles around the eyes [55]. Our results further support the notion that using a complex formulation consisting of five active materials in conjunction with retinol, a leading ingredient for wrinkle improvement, results in a more pronounced improvement in the appearance of both fine and deep wrinkles than that achieved with retinol alone. Although we only evaluated the effects over a period of 4 weeks because of limited study conditions, previous research has shown a consistent increase in the wrinkle improvement rate from 4 to 12 weeks or more, suggesting that this enhanced effect is likely to persist over the long term [27,54,55]. 

In this study, based on the hypothesis that *PPARGC1B* is related to the development of facial wrinkles, we showed a method of improving facial wrinkles in vitro, ex vivo, and in vivo using five materials that regulate *PPARGC1B*. However, the limited sample size and abbreviated duration are considered limitations of this study. Therefore, future longitudinal and in-depth investigations of the relationship between *PPARGC1B* and skin wrinkling are needed to elucidate the underlying molecular mechanisms.

## 4. Conclusions

The demand for managing skin aging, particularly facial wrinkles, which significantly contribute to cognitive age, is rising in modern society. Several strategies have been employed to delay or restore facial wrinkles, with continuous activity at the cellular level. In the field of skin research, consistent proposals are being made for methods to prevent and repair wrinkles by enhancing the function of mitochondria, the cellular energy source.

In this study, we highlight the potential of regulating *PPARGC1B*, which influences mitochondrial metabolism and biosynthesis, in modifying skin ECM components. After selecting five active materials that could enhance the expression of *PPARGC1B*, we confirmed their ability to strengthen and restore mitochondrial functions by measuring ΔΨm and ATP synthesis. Subsequently, we demonstrated that these active materials promote collagen synthesis at both the cellular level and in a 3D skin model. Particularly in the 3D skin model, we observed a collagen-synthesis-promoting effect by applying a cosmetic formulation containing the five selected active materials; this result indicated that the formulation affects 2D culture cells and promotes collagen synthesis when applied to actual human skin.

Finally, we conducted a human clinical trial using the LG formula. To further verify the effect of the LG formula, a representative wrinkle-improving ingredient, 0.1% retinol, was used as a control. The LG formula showed a superior rate of wrinkle improvement with regard to both fine lines under the eyes and nasolabial folds compared with the control group.

In conclusion, this study enhances our understanding of the facial wrinkle improving effect of a complex formulation of active materials that strengthen mitochondria by upregulating *PPARGC1B* expression. The study highlights the potential of five ingredients for antiaging products that should be effective for a wide range of facial wrinkles, from fine lines to deep wrinkles.

## Data Availability

The data that support the findings of this study are available from the corresponding author on request.

## Supplementary Materials

The following supporting information can be downloaded at: https://www.mdpi.com/article/10.3390/cimb46060302/s1, Figure S1: Effects of wrinkle improving materials on *PPARGC1B* expression. The materials that increased of decreased the expression of *PPARGC1B* in human dermal fibroblast, Hs68 treated with various candidates. Error bars represent the standard error of the mean. ** *p* < 0.05, * *p* < 0.1; Student’s *t*-test; Figure S2: Effect of two cream formulations on epidermal thickness in 3D skin equivalent.
