# Supplementary material for: Improvement in Facial Wrinkles Using Materials Enhancing PPARGC1B Expression Related to Mitochondrial Function
Source: Curr Issues Mol Biol. 2024 May 21;46(6):5037–51. doi: 10.3390/cimb46060302 (PMC11202557; doi:10.3390/cimb46060302)
Supplement: Supplementary file 1 [file cimb-46-00302-s001.zip › cimb-2983611-supplementary.pdf]

Supplementary Materials

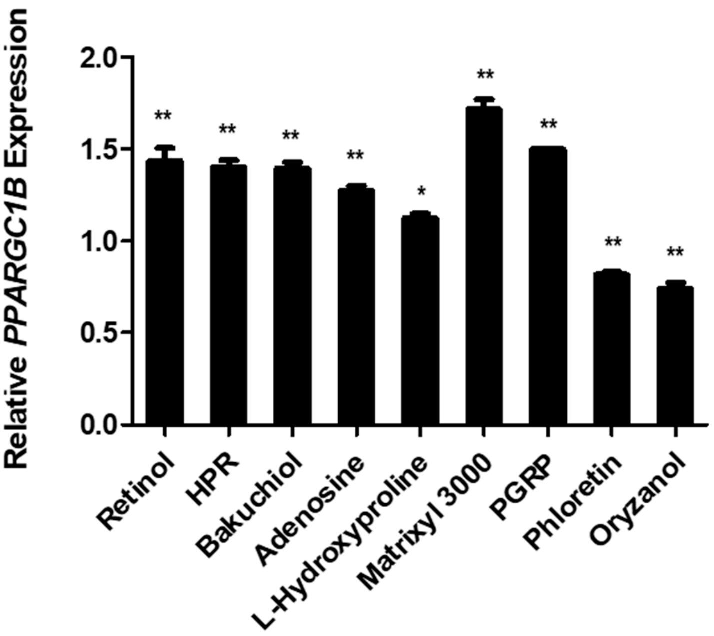

Figure S1. Effects of wrinkle improving materials on *PPARGC1B* expression. The materials that increased of decreased the expression of *PPARGC1B* in human dermal fibroblast, Hs68 treated with various candidates. Error bars represent the standard error of the mean. ** $p < 0.05$, * $p < 0.1$; Student's *t*-test.

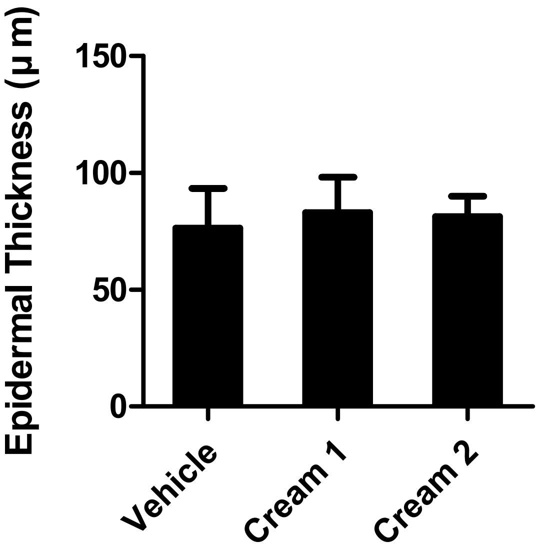

Figure S2. Effect of two cream formulations on epidermal thickness in 3D skin equivalent.
